# The Impact of Digital Health Transformation Driven by COVID-19 on Nursing Practice: Systematic Literature Review

**DOI:** 10.2196/40348

**Published:** 2022-08-30

**Authors:** Robab Abdolkhani, Sacha Petersen, Ruby Walter, Lin Zhao, Kerryn Butler-Henderson, Karen Livesay

**Affiliations:** 1 School of Health and Biomedical Sciences Science, Technology, Engineering, and Mathematics College Royal Melbourne Institute of Technology University Melbourne Australia

**Keywords:** digital health, COVID-19 pandemic, nursing informatics, nursing workforce

## Abstract

**Background:**

The COVID-19 pandemic has accelerated the uptake of digital health innovations due to the availability of various technologies and the urgent health care need for treatment and prevention. Although numerous studies have investigated digital health adoption and the associated challenges and strategies during the pandemic, there is a lack of evidence on the impact on the nursing workforce.

**Objective:**

This study aims to identify the impact of digital health transformation driven by COVID-19 on nurses.

**Methods:**

The online software Covidence was used to follow the Preferred Reporting Items for Systematic Reviews and Meta-Analyses (PRISMA) protocol. Relevant scientific health and computing databases were searched for papers published from January 2020 to November 2021. Using the 8D sociotechnical approach for digital health in health care systems, the papers were analyzed to identify gaps in applying digital health in nursing practice.

**Results:**

In total, 21 papers were selected for content analysis. The analysis identified a paucity of research that quantifies the impact of the digital health transformation on nurses during the pandemic. Most of the initiatives were teleconsultation, followed by tele–intensive care unit (tele-ICU), and only 1 (5%) study explored electronic medical record (EMR) systems. Among the sociotechnical elements, the human-related factor was the most explored and the system measurement was the least studied item.

**Conclusions:**

The review identified a significant gap in research on how implementing digital health solutions has impacted nurses during the COVID-19 pandemic. This gap needs to be addressed by further research to provide strategies for empowering the nursing workforce to be actively involved in digital health design, development, implementation, use, and evaluation.

## Introduction

In 2019, when the COVID-19 pandemic was declared worldwide, the delivery and organization of health care were propelled into an environment of rapid change. The change was associated with the deployment of staff, isolation of patients, protection of staff well-being, and public health education and initiatives to reduce or slow the transmission of the virus [1]. The pandemic has likely accelerated the adoption of digital health implementation [2]. Similarly, nurses providing care across a broad range of health care contexts, including community, primary health care, tertiary health care, and specialist services, such as aged care, are likely to be impacted by the uptake of digital technologies [3].

As the pandemic has not yet passed, it is expected that digital health will play an increasingly critical role in the future and even the postpandemic era due to the availability of various technologies and the urgent health care need for treatment and prevention [4]. The pandemic created a widespread and rapid adaptation to previously limited or unused forms of technology; this rapid adaptation has changed the ways clinicians and patients interact, for example, the now commonplace use of telehealth appointments rather than face-to-face appointments. Indeed, health care systems are investing much effort and focus on establishing long-term strategies to integrate digital health care models into routine practices and equip clinical professionals with knowledge and expertise to apply digital technologies efficiently [5].

The need for digital health implementation requires a transformation in nursing roles to acquire informatics-related skills to optimize and advance in this field in research, practice, and education. The pivotal contribution of nurses to the population’s health worldwide is undeniable. However, despite the necessity for increased digital health adoption in the health care system, the skills nurses need to obtain to participate in digital health implementation are not comprehensively understood. As stated in Wu’s study [6], the literature that discusses the need for nursing informatics skills was significantly increased since the COVID-19 outbreak (from January 2020 to August 2021), of which 36.7% emphasized informatics skills for nurses in clinical settings and 28.6% highlighted nursing informatics education. It is yet unclear how the COVID-19 pandemic is transforming the nursing professionals’ awareness of digital health—in other words, what skills nurses are required to achieve and how they can be trained during nursing education to efficiently act in digitally rich health care organizations.

Although digital health competencies for nurses were extensively outlined in the nursing informatics field [7], there are still uncertainties as to which technologies and to what extent nurses need to learn and work. Before the pandemic, digital health adoption was lacking due to numerous technical, organizational, environmental, behavioral, and operational challenges, whereas most of these issues were abandoned due to the pandemic priorities. However, despite the unprecedented expansion of digital health technologies during the pandemic, many countries worldwide were not sufficiently ready to utilize them due to these challenges [8]. Therefore, these issues need to be considered for adopting these initiatives to prepare the health care workforce for future pandemics [9].

The COVID-19 pandemic has transformed current digital health models to have an increased focus on interdisciplinary teams to co-design, develop, and implement digital health solutions [10]. Experienced nurses who received professional training in informatics can play a critical role in such teams. It is essential to understand which digital health technologies were established during the pandemic. Moreover, the environments in which they are used, the users interacting with those technologies, the communication between different tools and users, and the workflows and processes being performed all impact the data, information, and knowledge generated and the decisions made.

The digital health implementation experience during the pandemic indicates the need for effective planning to use these initiatives distinct from the prepandemic models. For example, it is critical to avoid disparities in accessing care when selecting digital health solutions [11]. These disparities include nurses who faced challenges accessing and using digital health technologies during the pandemic. To mitigate such inequities, nurses need to be educated and actively involved throughout the digital health pipeline. This study aims to answer the question “How did the digital health transformation driven by COVID-19 impact nurses?” The following questions address this overall question:

- Which digital health initiatives were implemented or expanded in health care during the COVID-19 pandemic?
- In what contexts were the digital health initiatives implemented or expanded?
- What issues influenced nurses’ experience of these digital health initiatives?
- What methods were used to identify these issues?
- What gaps were raised in these studies about nurses’ digital health experience during the pandemic that need to be considered?

## Methods

### Search Strategy

**Figure 1:**
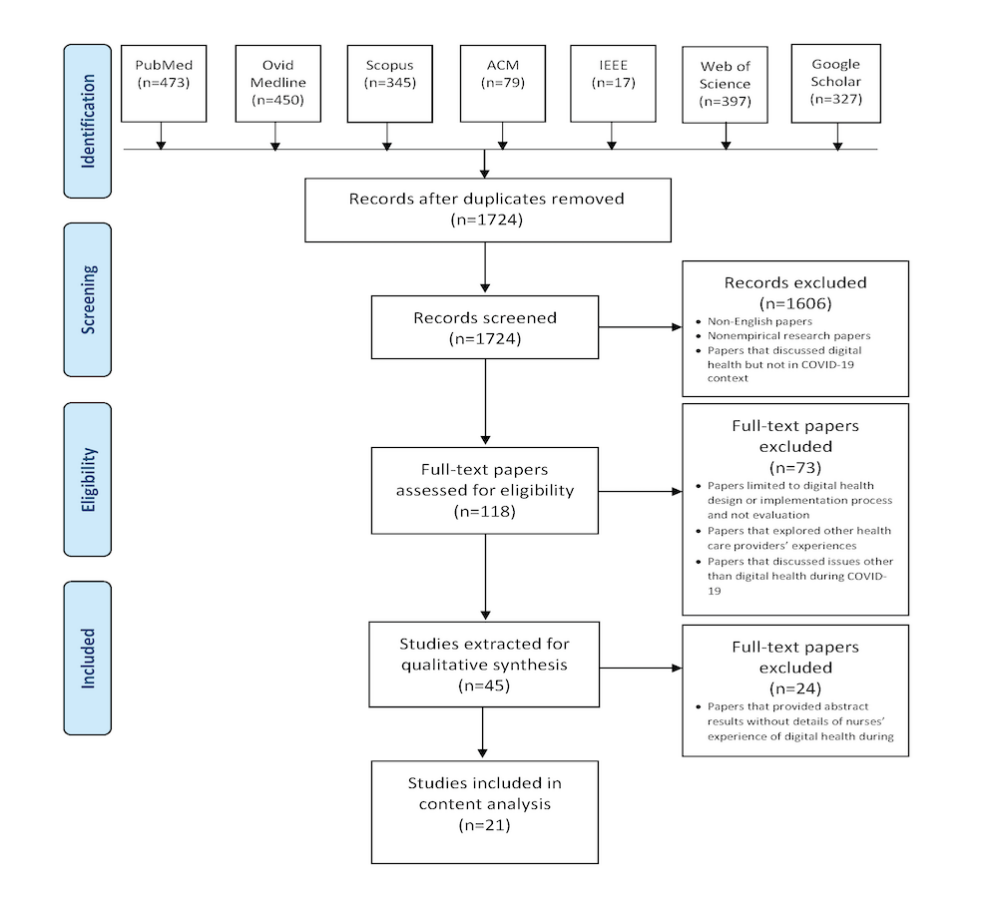
PRISMA diagram. ACM: Association for Computing Machinery; IEEE: Institute of Electrical and Electronics Engineers; PRISMA: Preferred Reporting Items for Systematic Reviews and Meta-Analyses.

A literature review was conducted using the online software Covidence following the Preferred Reporting Items for Systematic Reviews and Meta-Analyses (PRISMA) protocol [12] shown in Figure 1. The search concepts fell into 3 categories: COVID-19, digital health, and nursing. The concepts and their associated Medical Subject Headings (MeSH) and non-MeSH terms are described in Table 1. Cited and citing sources were double-checked to ensure no relevant items were missed.

The Boolean operator OR was used between the terms in each category, and then the operator AND was applied to combine the results by category. The search was applied to terms occurring in the title, abstract, or body text of full-text publications. The time frame selected was when COVID-19 was announced as a pandemic up to the time we conducted this study from January 2020 to November 2021. Scientific subscription databases searched contained both health and computing and information science databases, including PubMed, Ovid MEDLINE, Scopus, Web of Science, Institute of Electrical and Electronics Engineers (IEEE) Xplore, and Association for Computing Machinery (ACM) Digital Library. Google Scholar’s database was also searched using the same terms.

The search results were imported from Endnote into Covidence. The authors double-screened the titles and abstracts based on the eligibility criteria defined at the screening stage shown in the PRISMA flowchart. In the case of conflicts among the authors on a title or abstract exclusion, discussions were undertaken in regular meetings to reach a consensus. This approach was then applied to the selected abstracts to conduct the full-text review. The papers selected following double full-text review were accepted for the content analysis.

**Table 1:** Search terms.

Search concepts	MeSH^a^ terms	Non-MeSH terms
COVID-19	COVID-19	Coronavirus disease COVID-19 COVID-19 pandemic COVID-19 disease Global pandemic
Nursing	Nurse Telenursing Advanced practice nursing Evidence-based nursing Nursing education Nurse practitioners	Nurs* +: educators professional informatics informatician
Digital health	Artificial intelligence Augmented reality Big data Clinical decision support systems Computerized medical record Deep learning Electronic health record Electronic medical record Electronic record systems Health information exchange Machine learning Patient portals Remote sensing technology Social media Telemedicine Virtual reality	Connected health Digital health Digital medicine Digital therapeutics eHealth mHealth^b^ Mobile health Remote patient monitoring Remote monitoring Smart home Telehealth Telemonitoring Telerehabilitation Virtual care

^a^MeSH: Medical Subject Headings.

^b^mHealth: mobile health.

### Eligibility Criteria

Studies were eligible for review if they presented original research in the English language, discussed digital health initiatives implemented during the COVID-19 pandemic, and addressed nurses’ experience in using digital health during the pandemic. Exclusion criteria were considered at each stage of the PRISMA flowchart to exclude publications not relevant to this study.

### Content Analysis

A descriptive qualitative approach to content analysis [13] was used to describe the characteristics of the included studies that were relevant to the research questions outlined in the background. This approach is well suited for analyzing the multifaceted use of digital health in nursing practice in various health care contexts. Inductive reasoning was applied to investigate the literature to answer the study questions [14]. Next, the 8D sociotechnical model designed by Sitting and Singh [15] was selected to deductively analyze the selected studies and explore the interaction between digital health tools and technologies, individuals, and activities. This approach was applied to better understand the nurses’ experience of issues inherent in the design, development, implementation, use, and evaluation of digital health solutions during the pandemic. The findings were synthesized based on the 8 themes of the sociotechnical approach, including hardware and software computing infrastructure; clinical content; human-computer interface; people; workflows and communication; internal organizational policies, procedures, and culture; external rules, regulations, and pressures; and system measurement and monitoring. To ensure interrater reliability, 3 researchers undertook the content analysis independently.

## Results

### Characteristics of the Included Studies

A total of 21 studies met the inclusion criteria: 4 (19%) qualitative studies targeted nurses only [16-19], whereas the remaining 17 (81%) studies included nurses and other health care providers and did not differentiate nurses’ responses from others [20-36].

In addition, 13 (62%) studies explored users’ experience with digital health applications for COVID-19 control [16,17,19-22,26-30,35,36]. In comparison, 8 (38%) studies investigated the use of digital health to manage other acute and chronic conditions during the pandemic, including pediatric otolaryngology [23], alcohol management [18], cancer management [24], hemophilia [25], opioids [31], pediatric chronic pain [34], and mental [32], behavioral, and sexual health [33].

In terms of digital health services, 16 (76%) studies used teleconsultations in home care [16-25,30-34,36], 4 (19%) applied tele–intensive care unit (tele-ICU) [27-29,35], and 1 (5%) study focused on electronic medical records (EMRs) [26].

Regarding the type of health care setting, 10 (48%) studies were conducted in hospital settings [16,20,23,25-29,34,35], 8 (38%) studies were conducted in primary care facilities [17,19,21,24,30,31,33,36], 2 (10%) studies included both primary care and hospital organizations [18,32], and 1 (5%) study involved national nonclinical institutes [22].

The characteristics of the selected studies are shown in Table 2. The studies were sorted in alphabetical order.

**Table 2:** Characteristics of the selected studies (N=21).

First author/year	Type of digital health service	Study aim	Method
Arem/2021	Teleconsultations	Understanding the provider and survivor perspectives on the impact of telemedicine on cancer survivorship care during the pandemic	Of 607 health care providers, 273 (45%) were nurses. They were surveyed about their experience with telehealth.
Bavare/2021	Tele-ICU^a^	Studying how hybrid ward rounds could facilitate social distancing and maintain patient-centered care	Of 114 participants, 32 (28%) were nurses. They participated in a mixed methods study to evaluate the usability of a video conference platform implemented for interaction between the patient, the nurse, and the partial ICU team.
Belcher/2021	Teleconsultations	Understanding providers' experience of a 1-month otolaryngology teleconsultation pilot during the COVID-19 pandemic	Of 16 participants, 8 (50%) were nurses. They were surveyed about telehealth appointments that occurred during the study period.
Connolly/2021	Teleconsultations	Assessing provider perceptions of virtual care for mental health by comparing virtual (phone and telehealth) care to in-person care	Of 998 participants, 120 (12%) were nurses. They were surveyed about the quality and efficacy of virtual care versus face-to-face care with masks, the challenges of virtual care, and their willingness to continue virtual care in the future.
Esmaeilzadeh/2021	Electronic health records (EHRs)	Examining the impact of using EHRs on clinicians’ burnout in wards with COVID-19 cases	Of 368 participants, 147 (40%) were nurses. They were asked via a survey about cases of EHR-related burnout and usability challenges.
Franzosa/2021	Teleconsultations	Understanding clinicians' experience of COVID-19–related video visits	Of 13 participants, 3 (23%) were nurses. They were interviewed about the flexibility, benefits, and limits of teleconsultations, as well as factors to consider for future implementation of these services.
Garber/2021	Teleconsultations	Identifying the difference between the COVID-19 telehealth training levels for clinicians and the perceived usefulness, self-efficacy, knowledge, satisfaction, and frequency of actual use of telehealth	Of 224 participants, 47 (21%) were nurses. They were surveyed on the usefulness, self-efficacy, knowledge, use, and satisfaction of telehealth based on the telehealth training provided.
Gilkey/2021	Teleconsultations	Characterizing primary care professionals’ recent use and attitudes of adolescent telehealth during COVID-19 and their support of offering telehealth after the COVID-19 pandemic	Of 1047 participants, 123 (12%) were nurses in a national survey of primary care professionals on their experience of teleconsultations with adolescents.
Hughes/2021	Teleconsultations	Exploring nurses’ experiences of utilizing virtual care and remote working during COVID-19 to identify what elements could be implemented into a recovery model following the pandemic	In total, 17 operational lead nurses and 31 nurses at different levels of seniority were interviewed (semistructured) on the advantages and disadvantages of telehealth and whether they would support telehealth after the pandemic. Interviews of the first cohort (n=17, 100%) informed the interviews of the second cohort (n=31, 100%).
Hunter/2021	Teleconsultations	Understanding the change of service delivery with opioid treatment programs through the use of teleconsultations during COVID-19	Of 20 participants, 3 (15%) were nurse practitioners. Semistructured phone interviews were conducted on their experience of using teleconsultations and quality implications.
James/2021	Teleconsultations	Exploring the experiences of primary health care nurses in the use of teleconsultations during COVID-19	All 25 (100%) participants were nurses. Semistructured phone interviews were conducted on their preparedness and experience of telehealth.
Killackey/2021	Teleconsultations	Understanding how pediatric pain care clinics operated during COVID-19 and the uptake of virtual care	Of 151 participants, 17 (11%) were nurses. Two online cross-sectional surveys on experience of virtual care were conducted.
Mohammed/2021	Teleconsultations	Exploring the use of virtual visits among primary care providers during COVID-19 and anticipated use postpandemic, understanding user perceptions of the available support tools, and identifying factors that influence success and challenges to adoption and incorporation of virtual visits	Of 200 participants, 37 (19.5%) were nurse practitioners. A descriptive cross-sectional survey of their experience with virtual visits was conducted.
O’Donovan/2020	Teleconsultations	Exploring patient and health care provider experience of telehealth in the European Haemophilia Comprehensive Care Centre during COVID-19	Of 21 health care provider participants, 9 (43%) were nurses. A systematic evaluation of patient surveys and health care provider surveys was performed, and a comparison of in-person appointments and teleconsultations was made.
Park/2021	Teleconsultations	Assessing the satisfaction of patients and medical staff with telemedicine used during the 17-day hospital outpatient/emergency department (ED) closure due to COVID-19	Of 155 medical staff participants, 100 (64.5%) were nurses. Surveys via text were conducted to assess their satisfaction with and experience of teleconsultations.
Pilosof/2021	Tele-ICU	Examining the implementation of telemedicine in COVID-19 ICUs	Of 30 participants, 3 (10%) were nurses. Semistructured interviews were conducted with medical staff, telemedicine companies, and architectural design teams on the use of telemedicine.
Ramnath/2021	Tele-ICU	Developing, implementing, and evaluating a novel tele-ICU program during the COVID-19 pandemic	Of 27 participants, 10 (37%) were nurses. Performance metrics were collected, and surveys of staff perceptions of tele-ICU were conducted.
Safaeinili/2021	In-patient telemedicine	Assessing the acceptability and effectiveness of in-patient telemedicine during the COVID-19 pandemic	Of 15 participants, 5 (33.3%) were nurses. Semistructured interviews were conducted with nurses and attending and resident physicians on the use of telemedicine.
Searby/2021	Teleconsultations	Examining the experiences of alcohol and other drug (AOD) nurses transitioning to telehealth during COVID-19	All 19 (100%) participants were nurses. Semistructured interviews were conducted with AOD nurses from Australia and New Zealand on the use of telehealth.
Silva/2021	Teleconsultations	Reporting on monitoring of patients by nursing students who volunteered at COVID-19 clinics with telehealth duties.	All 17 (100%) participants were nurses, including nursing students (n=14, 82%) and nursing professors (n=3, 18%). A descriptive experience report was prepared on phone monitoring 1400 patients, ~80 cases per day.
Srinivasan/2020	Teleconsultations	Assessing rapid transformation to video consultations at an academic medical center during COVID-19	Of 53 participants, 4 (7.5%) were nurses. Semistructured interviews of perspectives on video visit acceptability were conducted.

^a^ICU: intensive care unit.

### Sociotechnical Elements of Using Digital Health in Nursing Practice

The selected studies were synthesized according to the sociotechnical approach. Table 3 shows the frequency of the 8 dimensions of the sociotechnical approach for using digital health technology systems in the selected studies.

As shown in Table 3, none of the selected studies addressed all the 8 dimensions of the sociotechnical approach when exploring nurses' use of digital health during the pandemic. Among the 8 dimensions, the people-related aspect was the most frequently discussed item, studied by 17 (81%) papers, whereas system measurement and monitoring were mentioned in only 2 (10%) papers. Details of each dimension are addressed in Table 4.

**Table 3:** Frequency of the sociotechnical themes in the included studies (N=21).

Author	Sociotechnical theme addressed (yes/no)							
	Hardware and software computing infrastructure	Human-computer interface	Clinical content	People	Workflows and communication	Internal organizational policies, procedures, and culture	External rules, regulations, and pressures	System measurement and monitoring
Arem	Yes	Yes	Yes	Yes	No	Yes	Yes	No
Bavare	Yes	Yes	No	Yes	Yes	Yes	No	No
Belcher	Yes	No	No	No	Yes	No	No	No
Connolly	Yes	Yes	No	Yes	Yes	No	No	No
Esmaeilzadeh	No	Yes	Yes	Yes	No	Yes	Yes	Yes
Franzosa	Yes	No	No	Yes	Yes	No	No	No
Garber	No	No	No	Yes	No	No	No	No
Gilkey	Yes	No	Yes	Yes	No	Yes	No	No
Hughes	Yes	No	No	Yes	No	No	No	No
Hunter	Yes	No	No	No	Yes	Yes	No	No
James	No	No	No	Yes	No	Yes	No	No
Killackey	Yes	Yes	No	Yes	Yes	Yes	Yes	No
Mohammed	Yes	No	No	Yes	Yes	Yes	No	No
O’Donovan	No	No	No	No	Yes	Yes	No	No
Park	Yes	No	No	Yes	Yes	No	No	No
Pilosof	Yes	Yes	No	Yes	No	No	No	No
Ramnath	No	No	No	Yes	No	No	No	No
Safaeinili	No	No	No	Yes	Yes	No	No	No
Searby	Yes	No	No	Yes	Yes	No	No	No
Silva	No	No	No	Yes	Yes	Yes	No	No
Srinivasan	Yes	No	No	Yes	Yes	No	Yes	Yes

**Table 4:** Considerations for sociotechnical elements of digital health practice in nursing addressed in the literature.

Sociotechnical themes	Considerations
Hardware and software computing infrastructure	Unreliable internet access [18,21,24] Variable audiovisual quality and internet connection [16,21,29,33,34,36] Lack of appropriate video and audiological mechanism to perform a physical examination remotely [18,20,23,30-34,36] Limited visibility in tele-ICU^a^ cameras [35]
Human-computer interface	Screen freezing and audio delays [20,24,34] Alarm interference [29] Difficulties in video platform usability [32] High volume of data entry [26] Multiple visual communication on segregated screens [35]
Clinical content	Lack of interpretation services [24,33] Need for a data analyst to extract meaningful insights from data [26]
People	Limited training on using telehealth and in-patient telemonitoring [17,19,21,24,28,30,32-34,36] Concerns about the privacy of patients’ information [21,24,33-36] Concerns about missing something important about patients [16-18,24,36] Lack of teaching during rounds and lack of situational awareness [29] Poor leadership support for telehealth [21,32,34] Increased burnout in using EMRs^b^ due to lack of training [26] Lack of trust in discussing sensitive topics via video visits [30] Lack of provider’s adoption of telehealth due to training as written instructions without actual telehealth use [22] Fatigue from conducting a large number of video visits [16,20,36] Feelings of disconnection and lack of social interaction with patients [16,18,28,35] Need for the implementation team to perform innovative change in implementing tele-ICU in critical care [27]
Workflows and communication	Inconsistency in team communication and presence at the bedside [28,29] Concern about full responsibility of clinicians for connectivity that limits the volume and efficiency of telehealth visits [23] Difficulties in scheduling procedures [28,32] Need for streamlined video integration into clinical workflows [21,32] Challenges in setting up a large number of patient portal accounts [30] Challenges when needing to rapidly consent patients [30] Need for extra time to communicate and coordinate visits [21,25,28,31] Lack of local information technology (IT) support and communication [34,36] Lack of integration between telehealth visits and EMRs and need for roles to support these integrations [21] Miscommunication [20] Nursing being the only profession left to work on-site during the transition to telehealth [18] Constant updates for actions [19]
Internal organizational policies, procedures, and culture	Difficulty capturing reimbursement for services [21,24,25,33,34] Lack of integrated approach to implementing tele-ICU across the setting [29] Need for clear regulations and policies about clinicians’ responsibilities when making errors using technology [26] Lack of preparedness and strategies on which platform to use for video visits [17,31] Constraints of the funding models in health care settings [17] Lack of equipment for using telehealth services [19]
External rules, regulations, and pressures	Issues with multijurisdictional licensure [24,36] Concerns about complying with legal standards [24] Litigation concerns [24] Need for unified standards for order entry and reporting among hospitals [26] Limited licenses for video visit platforms [34]
System measurement and monitoring	Need for regular assessment of technology effectiveness [26] Need for a systematic approach to measure workplace burnout in using technology [26] Need for new digital data sources and comprehensive remote monitoring systems to continue and scale up the services and make them ongoing part of clinical practice [36]

^a^ICU: intensive care unit.

^b^EMR: electronic medical record.

#### Hardware and Software Computing Infrastructure

This dimension focuses on the hardware and software components of digital health technologies, such as machines, devices, storage, network, and power required to run the applications. Most studies addressed audiovisual issues with variable quality in telehealth services, which led to difficulties in undertaking physical exams virtually [18,20,23,30-34,36]. An unreliable internet connection [16,21,29,33,34,36] and lack of access [18,21,24] also delayed the communication between nurse and patient in these services. Limited connectivity in the network caused poor visibility when using tele-ICUs, which resulted in a noncomprehensive picture of the patients, wards, and beds [35].

#### Human-Computer Interface

The interface includes aspects of the technology with which the user can interact. It contains any issues that users find in using the systems. The studies on telehealth reported that screen freezing, audio delays [20,24,34], and alarm interference [29] impacted their interaction with patients during the virtual visits. As health care organizations rapidly implemented telehealth during the pandemic and workflows highly increased, there was a heavy load of data entry and a lack of time for it and documentation [26]. Moreover, the implementation of various platforms that the nurses were unfamiliar with brought usability challenges [32]. Connecting via segregated screens led to multiple visual communications that added a burden to the workforce [35].

#### Clinical Content

The lack of interpretation services was challenging to nurses and other health care providers [24,33]. The new digital health services provide new information and insights to the health care workforce and require further interpretation. Of the 21 studies, 1 (5%) study reported a need for a data analyst to extract meaningful information and insights from the large volume of data collected from various tools to manage people’s health during the pandemic [26].

#### People

Most of the studies addressed the lack of training in using new home telehealth and in-patient telemonitoring initiatives and EMR use, which led to burnout as well as fatigue from conducting a large number of virtual visits [16,17,19-21,24,26,28,30,32-34,36]. There were concerns among nurses and other clinicians that they might miss important things, such as emotional interactions, when the visits were conducted virtually [16-18,24,36]. The respondents felt disconnected and did not have the social interaction they usually would during in-person visits [16,18,28,35]. Being connected through various devices and networks, many reported concerns about patients’ privacy [21,24,33-36] and felt there was not enough trust to discuss sensitive topics virtually [30]. Poor support from leadership in providing training and improved situational awareness was another challenge to using digital health efficiently [21,29,32,34]. The studies also showed that written instructions on using different technologies without proper practical testing do not enhance providers’ adoption of the digital health [22]. Nurses were not involved during digital health implementation to understand the process and raise the issues nurses encounter in incorporating digital health into their workflow [27].

#### Workflows and Communication

The pandemic has interrupted clinical workflows due to increased demands for health care. The increased use of digital health has added another layer of interruption. Inconsistency in team communication [28,29]; difficulties in scheduling procedures [28,32], setting up different accounts, and obtaining various consents [30]; and constant updates [19] were among the issues the studies raised about workflows. The nurses were concerned about having the full responsibility for connectivity [23], which required extra time to communicate and coordinate visits [16,20,23,26], in turn limiting the number and efficiency of telehealth visits. Therefore, the nurses requested streamlined video integration into clinical workflows [21,32]. Nurses also raised disconnection [20] and lack of support from information technology (IT) support teams when using digital health services [34,36].

#### Internal Organizational Policies, Procedures, and Culture

The shortage of funding and reimbursement models was a significant internal issue that impacted the implementation of digital health use during the past 2 years [17,21,24,25,33,34]. The studies reported a lack of strategies and policies for integrating different tools across health care settings, workflows, training, and preparedness [17,29,31]. In addition, once the pandemic escalated, limited equipment was available for telehealth services [19]. There was an absence of policies on integrating different platforms and clarity about accountability for medical errors when using digital health services [26].

#### External Rules, Regulations, and Pressure

As many telehealth services were implemented, concerns arose around the lack of compliance with legal standards for data sharing, multijurisdictional licensure, and litigation [24,34,36]. The studies emphasized the need for unified standards for data collection, documentation, usage, and sharing among health care settings [26].

#### System Measurement and Monitoring

This element was the least explored sociotechnical dimension, with only 2 (10%) papers presenting relevant data in this area. They reported a lack of regular assessment of technology effectiveness. They suggested developing a systematic approach to measure burnout and usability and the need for new digital data sources and strategies to scale the initiatives to be integrated into the workflow [26,36].

## Discussion

### Principal Findings

This literature review identified that the sociotechnical aspects of digital health in nursing practice are not widely investigated by the literature examining the impact on nursing practice due to the digital health transformation driven by COVID-19. Despite their recognized importance for efficient interaction between nurses, the technology and process within the health care environment were largely not addressed. This suggests that there are gaps in how the interaction with digital health innovations contributes to nurses’ practice to provide efficient care and ensure patient safety and the quality of care. The results showed that the clinical participants mainly discussed patient experiences of digital health, not their own experiences. This indicates a lack of emphasis on understanding nurses’ struggles within their workflows.

Of the 21 studies selected, only 4 papers examined the nurses’ views and experiences of digital health during the pandemic. Undoubtedly, implementing digital health innovations requires a multidisciplinary team, which may explain the multidisciplinary approach of many of the papers examined. However, there needs to be a dedicated examination of the issues nurses face in using digital health during their routine practice in different care settings to identify nursing-specific needs.

Teleconsultations were the most used type of digital health during the pandemic, as evidenced in 16 studies. The high demand for social distancing and the reduction in in-person visits shifted clinic appointments to the virtual model. The findings showed that the health care settings that had already implemented telehealth services and needed to expand them during the pandemic were more confident, satisfied, and less challenged than those that implemented them for the first time in the pandemic. Overall, the studies reported positive attitudes among nurses toward applying telehealth consultations, such as satisfaction, improved communication with patients [23,30,33], improved productivity and quality of care [16,32], and enhanced flexibility in the workflow [16].

The other digital health service applied during the pandemic was tele-ICU, explored in 4 studies, to keep the optimal distance between the control team and clinical providers within acute care departments. The studies showed overall satisfaction with the service that improved the workflow, teamworking, and patient-centered care [27-29,35].

Surprisingly, only 1 study investigated the issues related to the EMR system during the pandemic. EMRs are 1 of the tools that might have been implemented by most of the health care settings before the other digital health services in the prepandemic era. The study explored the causes of EMR-related burnout during the pandemic as the workload and documentation suddenly increased. In contrast to the positive outcomes of virtual visits reported in the studies discussed earlier, the burnout of EMR utilization mainly was reported due to a lack of face-to-face interactions [26].

Regarding disease treatment and monitoring, 13 studies used digital health exclusively for COVID-19, while 8 reported transitioning to telehealth to manage other conditions during the pandemic. Considering the opportunities and challenges expressed in the studies, the digital health transformation requires strategies to overcome the barriers and provide ways to sustain the initiatives for integration with routine nursing practice.

### Gaps in Sociotechnical Elements of Digital Health in Nursing Practice

The synthesis of the elements of the sociotechnical approach in the literature showed that the rise and acceleration of digital health during the COVID-19 pandemic era has posed challenges regarding usability, functionality, and transformation. The sociotechnical elements were not thoroughly investigated in the literature, whereas the implementation and use of digital health services require interaction between the human, technology, and process-related factors shown via the sociotechnical approach elements to provide efficient and quality care.

The predominant consideration discussed in the literature was the people factor, which highlights the need to focus further on the nursing workforce. As most digital health studies concentrate on the technology, intervention, or outcome, the people factor, people’s needs, and their competencies are usually overlooked. System measurement and monitoring was the least explored element, indicating that despite the massive implementation of digital health services, there is little research on evaluating effectiveness and plans for ongoing use of services and nurses’ roles in these evaluations.

The literature showed that nurses are less likely to adopt the appropriateness of telehealth services [24] and to use secure messaging in providing virtual care [21]. Anxiety was the biggest challenge for nurses transitioning to telehealth, because moving toward telehealth would reduce patients’ and nurses’ engagement to interact with each other [18]. Nurses reported that the nursing care provided face to face cannot be easily replicated in a telehealth setting due to the type of care provided and the patient group’s needs [16]. Telehealth is a barrier to providing basic nursing skills, such as reassuring a patient [18]. Nurses perceived that their exposure to patients stays at a similar level after implementing in-patient telemedicine [28].

Lack of training has been identified as 1 of the core causes hampering the usability of the digital health system. Further, the literature has identified the connection between poor usability and varying levels of trust in using new technology in privacy protection [21,24,30,33-36]. Therefore, the solution lies in ensuring safe implementation training embedded in the routine training of nurses from undergraduate courses to postgraduate training and continuous professional training. Dedicated training in health informatics provides nurses with in-depth knowledge and skills to become comfortable with technological changes. It translates more robust safeguards to health information used in the digital health platform [37].

Although the telehealth services were perceived as an acceptable solution to increase access to care services during the pandemic [17] and were rapidly deployed during the pandemic, insufficient resources to implement telehealth at a large scale lowers the reimbursement and nurses’ motivation to use the services [24]. The prepandemic studies have addressed the reimbursement challenge as the most significant barrier to scaling up pediatric telehealth programs. The participants reported that reimbursement was a major barrier to the widespread use of telemedicine before the pandemic [31]. However, the payer reimbursement rapidly expanded during the pandemic [33]. As telehealth services are increasingly adopted in primary care, funding for general practice nurses needs to continue to facilitate better use of their roles [17].

There was a vast difference among nurses in various specialties in the perceived quality of care delivered via telehealth. This emphasizes the need for training in virtual physical examinations for different diseases [32]. Similarly, Esmaeilzadeh et al [26] found that the leading cause of EMR-related stress is insufficient training in the use of technology. However, receiving education as written instructions only was deemed the equivalent of having no education in using telehealth and did not enhance the adoption of these services [22]. There was insufficient training on how staff should use and support video visits conducted for the first time [36]. Limited telehealth education in health care professional programs might result from a lack of faculty expertise, technology, or opportunities for clinical experience in telehealth [22]. Nurses’ preparedness to use telehealth depends on the availability of technology and the skillset [17]. Moreover, interpretive services need to be embedded in future telehealth implementations to facilitate communication and improve nursing practice [23].

The other most challenging issues were the usability issues of technology and complicated workflows and communication. Clinicians had challenges deciding which infrastructure, logistics, and platforms to use [34]. The technological challenges were often linked to financial barriers [34]. Lack of adequate resources and the need for more time to conduct teleconsultations were the main challenges reported by O'Donovan et al [25].

System-level challenges exist in navigating payment models offered by different insurance companies, licensure, and litigation issues [24]. Nurses raised concerns about poor phone connectivity that hampered communication and jeopardized patient safety [29]. They felt that telehealth consultations increase their responsibility for connectivity and intake, which would severely limit the volume and efficiency of telehealth services [23]. For example, in tele-ICU services, nurses were frustrated about staying away from the bedside to overcome communication barriers. This increased the need for another nurse to fulfil the needs of critically ill patients [29].

A significant drawback of virtual visits is the inability to conduct physical examinations remotely [32]. There are concerns about not getting a holistic picture of the patient’s status in video visits due to technological and connection issues [20,30]. The inability to adequately assess the patient’s situation due to audiovisual issues reduces the quality of care [31]. There was an increased risk of nurses being unable to visually assess the patients and having difficulties assessing risks, such as domestic violence [18]. The findings showed that those with more EMR experience were more likely to be concerned about patients’ increased demand for virtual care, which is not integrated into EMR workflows [21]. Therefore, more investment is required in the internet infrastructure and access [34]. Effective support processes, such as change management strategies for integrating EMRs and virtual care services, will improve nurses’ practices in providing efficient care [21].

These challenges are not surprising. The rapid adoption of new digital health technologies at short notice in complex health care systems is an economically challenging and time-consuming process [2]. However, this justifies the need for more research in this area. Understanding these challenges can help better prepare nurses for using digital health in clinical practice and not stretch already exhausted systems.

### Comparison With Prior Work

Several literature review studies have been published since the pandemic began that explore the use of digital health in this context [5,8,38-40]. However, these studies have discussed digital health in general and have not focused on nursing practice. This study is the first of its kind that reviewed original research conducted during 2020-2021, focusing on nurses’ digital health experience. It used the 8D sociotechnical approach to comprehensively address the challenges related to the design, development, implementation, use, and evaluation of digital health initiatives that nurses confront in their practice in complex, real-world settings.

### Limitations and Future Work

As the scope of this review was limited to the past 2 years, it did not cover the research conducted before the pandemic on nurses’ experience with digital health. Moreover, it was limited to English papers and original research. Due to the pressure on the health care systems, nursing practice in many regions might not have researched the nurses’ experience and outcomes during the pandemic or their findings might have been published as white papers or industry reports that were excluded from our screening.

### Conclusion

This literature review studied the role and engagement of nurses in the digital transformation of health during the first 2 years of the COVID-19 pandemic. Despite the rapid growth and adoption of digital health [41], as the largest group of health care providers, nurses remained passive users of these technologies. The sociotechnical elements showed a lack of assessing nurses' interaction with and use of digital health. Despite overwhelming evidence suggesting digital health care improves efficiency and equity, many of the studies reviewed highlighted concerns about adopting digital health. Examples included disrupted workflows, lack of regulatory support, and ongoing system monitoring. It is well known that the health care industry is always slow in implementing new technology, which is often a safety-driven decision or due to a lack of supportive evidence [42]. Further, it usually takes a long time to validate and integrate digital health into the existing health services due to the complexity of this process. Recognizing the impact of the health service system and policy on digital health, all stakeholders need to be involved so their needs and interests can be incorporated into the digital health policy development and planning. Future work can explore ways in which nurses can be actively engaged in the design, development, implementation, and efficiency assessment of digital health within health care systems.

## Abbreviations

EMR: electronic medical record
ICU: intensive care unit
MeSH: Medical Subject Headings
PRISMA: Preferred Reporting Items for Systematic Reviews and Meta-Analyses
